# Alpha2 Macroglobulin-Like Is Essential for Liver Development in Zebrafish

**DOI:** 10.1371/journal.pone.0003736

**Published:** 2008-11-17

**Authors:** Sung-Kook Hong, Igor B. Dawid

**Affiliations:** Laboratory of Molecular Genetics, Eunice Kennedy Shriver National Institute of Child Health and Human Development, National Institutes of Health, Bethesda, Maryland, United States of America; Katholieke Universiteit Leuven, Belgium

## Abstract

**Background:**

Alpha 2 Macroglobulin family members have been studied extensively with respect to their roles in physiology and human disease including innate immunity and Alzheimer's disease, but little is known about a possible role in liver development loss-of-function in model systems.

**Principal Findings:**

We report the isolation of the zebrafish *α2 macroglobulin-like* (*A2ML*) gene and its specific expression in the liver during differentiation. Morpholino-based knock-down of *A2ML* did not block the initial formation of the liver primordium, but inhibited liver growth and differentiation.

**Significance:**

This report on A2ML function in zebrafish development provides the first evidence for a specific role of an A2M family gene in liver formation during early embryogenesis in a vertebrate.

## Introduction

The α2 macroglobulin (A2M) family includes conserved blood proteins with multiple functions, especially as protease inhibitors [1], [2], [3], [4]. While A2M is produced in the liver, multiple patterns of tissue distribution and various functions, including a link to Alzheimer's disease, have been reported for different A2M family members [5], [6], [7], [8], [9]. Complete A2M deficiency has not been reported in humans, but mice with targeted mutations in the A2M or the related murinoglobulin gene are viable and fertile, with susceptibility to acute pancreatitis as the most apparent phenotype [10], [11]. In *Xenopus laevis,* the A2M-related genes *endodermin* (*EDD*) and *Panza* are expressed in liver and in other endodermal derivatives [12], [13], but developmental aspects of A2M family function in liver development have not been reported in this or any other species. Here we report the isolation of the zebrafish *alpha-2 macroglobulin-like (A2ML*) gene, and show that it has a role in liver development in this organism. Morpholino-based knock-down of *A2ML* does not block the initial formation of the liver primordium, but growth and development of the liver is disrupted largely by reduced cell proliferation, without observable defects in pancreas and intestine development. Thus we present evidence for the role of an A2M family gene in liver formation during embryogenesis.

## Results and Discussion

### Liver specific expression of zebrafish *A2ML*

**Figure 1:**
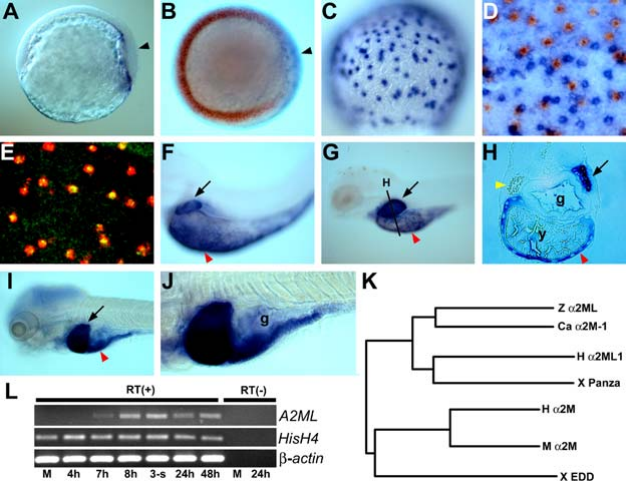
Expression and phylogenic tree analysis of zebrafish A2ML. A–B. Lateral (A) and dorsal view (B) of restricted expression of *A2ML* under the embryonic shield at 70% epiboly. (B) is double labeled with *vent* (red). Arrowheads in A and B point to the embryonic shield. C–E. 80% epiboly stage. Lateral view of *A2ML* expression in the nucleus of YSL cells. (D) Two color in situ hybridization with *cas* (blue) and *A2ML* (red). (E) Fluorescence in situ hybridization with *tfa* (green) and *A2ML* (red). F. Lateral view of *A2ML* expression in the liver at 3.5dpf. G–H. Restricted expression of *A2ML* in liver and yolk (G) and transverse section (H) at 4.5dpf. Yellow arrowhead points to *A2ML*-negative pancreas rudiment. I–J. Lateral view of *A2ML* expression at 7dpf. K. Phylogenic tree of A2M family. Ca, carp; H, human; M, mouse; X, Xenopus, Z, zebrafish. See Supplementary Material for accession numbers. L. RT-PCR analysis of *A2ML* in development; *Histone H4* and β-*actin* were used as controls. Arrows in F–I indicate developing liver, and red arrowheads in F–I point to yolk. g, gut; y, yolk.

A partial sequence for the zebrafish *α2-macroglobulin like gene (A2ML)* was listed under GenBank accession number BC125959 (IMAGE: 6996802), and is localized on chromosome 15 in the Sanger Center Zebrafish Genome Data base (Scaffold Zv7_scaffold1487 http://www.ensembl.org/Danio_rerio/). To extend this sequence we performed the RACE procedure, obtaining full-length *A2ML* cDNA (accession number EU689051). Zebrafish A2ML contains three domains that are conserved in the vertebrate A2M family (Fig. S1A–D). Zebrafish A2ML is more closely related to human A2ML and to *Xenopus* Panza than to the prototype A2M of human, mouse and *Xenopus* (Fig. 1K). Expression of *A2ML* begins in the yolk syncytial layer (YSL) beneath the dorsal shield in the zebrafish gastrula, as verified by two color in situ hybridization with *vent* as a ventral marker [14], [15] (Fig. 1A,B). Zygotic expression of *A2ML* was confirmed by RT-PCR (Fig. 1L). *A2ML* continues to be expressed in the YSL during mid gastrulation, as confirmed by double-label staining with *Casanova (cas)* [16] and *transferrin a (tfa)* [17], [18] as endoderm and YSL markers, respectively (Fig. 1C–E). Expression of *A2ML* in the liver starts around 3.5 days post fertilization (dpf) (Fig. 1F), and continues at later stages, in addition to expression in the epithelium surrounding the yolk (Fig. 1G,H), while the pancreas rudiment is negative at all stages tested (yellow arrow in Fig. 1H). By 7 dpf, the liver continues strongly positive while the gut also shows expression of *A2ML* (Fig. 1I,J). These data indicate that A2ML, a member of the A2M family, is most strongly expressed in the developing liver during early zebrafish development.

### A2ML is not required for the initiation of liver formation

**Figure 2:**
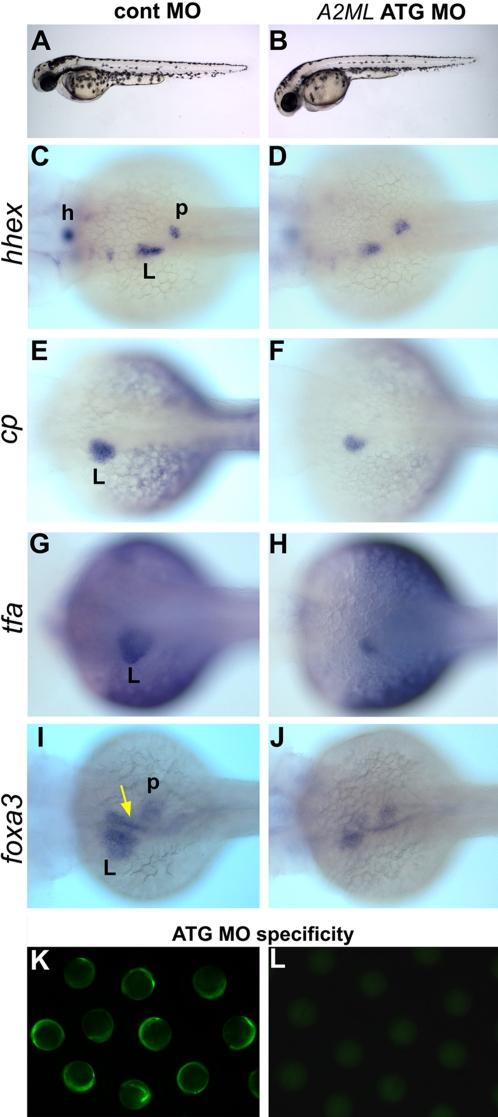
*A2ML* knock-down phenotype. *A2ML* ATG MO-injected embryos were at 50 hpf (A–J); embryos in the MO specificity test were at the 3-somite stage (K–L). A–B. Lateral view of live images of control and *A2ML* ATG MO-injected embryos. C–J. Dorsal views of whole mount in situ hybridization with *hhex* (C–D), *cp* (E–F), *tfa* (G–H), and *foxa3* (I–J) of cont MO (C, E, G, I) and *A2ML* ATG MO (D, F, H, J) injected embryos. A yellow arrow in (I) points to the developing hepatopancreatic duct. K–L. An *A2ML-GFP* fusion construct was expressed in 95% (*n* = 40) of embryos after coinjection with control MO, while coinjection of *A2ML* ATG MO blocked expression of the fusion construct in 95% (*n* =  55) of embryos. h, heart; L, liver; p, pancreatic primodium.

To study A2ML function in development, we used two anti-sense morpholinos (MO) to inhibit its expression. One MO is complementary to the 5′-UTR (UTR MO) while the other targets the translation start area (ATG MO). Fig. 2 compares embryos injected with *A2ML* ATG MO or control MO. Knock-down embryos exhibit largely normal development (90%, *n* =  87), similar to embryos injected with cont MO (95%, *n* =  33) (Fig. 2A,B). Because of the expression of *A2ML* in endoderm we tested for early effects of the MO on liver and pancreas development, using markers such as *hhex* [19], *ceruloplasmin (cp)* [20], *tfa*, and *foxa3* [21] (Fig. 2C–J). At 50 hpf, expression of these markers in liver is partially reduced by *A2ML* MO, while the pancreatic primordium is normal. Specificity of this MO was tested using an *A2ML*-GFP fusion construct, whose expression is effectively blocked by the morpholino (Fig. 2K,L). We also tested *hhex* and *cp* expression at 24 hpf and detected no changes in liver and pancreatic primodium development (data not shown). These results indicate that the development of the liver and pancreas is initiated normally in *A2ML* ATG MO-injected embryos, but that liver formation begins to be affected after 2 days of development.

### A2ML is essential for liver and gut, but not pancreas formation

**Figure 3:**
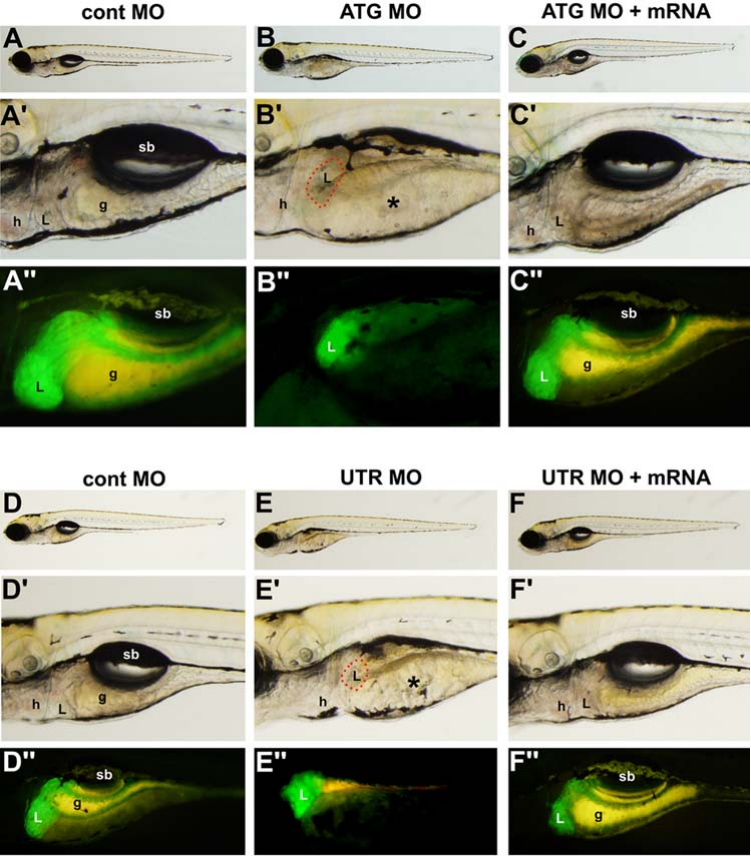
*A2ML* MOs affect liver formation. Embryos from the *ef1a-GFP* transgenic line are shown at 5dpf. A–A″. cont MO-injected embryos (95% normal, *n* = 30). B–B″. *A2ML* ATG MO-injected embryos showed reduced liver in 90% (*n* = 45) of cases. C–C″ Rescue of *A2ML* ATG MO phenotype by co-injection with *A2ML* mRNA (80% rescued, *n* = 45). D–D″. Cont MO-injected embryos (99% normal, *n* = 35). E–E″. *A2ML* UTR MO injected embryos (98% defect, *n* = 55). F–F″. Rescue of *A2ML* UTR MO phenotype by co-injection with *A2ML* mRNA (90% rescued, *n* = 47). See Experimental Procedures for injection levels. Red outlines in B′ and E′ represent the small livers remaining in these embryos. Asterisks in B′ and E′ identify yolk. g, gut; h, heart; L, liver; sb, swim bladder.

Knock-down of *A2ML* expression led to specific deficits in liver formation that became more pronounced with time, without causing widespread developmental defects. These results were confirmed with the aid of the transgenic line *Tg(XlEef1a1:GFP)* that allows visualization of intestinal organs [22]. Injection of either UTR or ATG MO led to a strong reduction in the size, albeit not complete disappearance, of the liver in 5 dpf embryos (Fig. 3). Importantly, this defect could be rescued by co-injection of the MOs with *A2ML* mRNA (Fig. 3).

**Figure 4:**
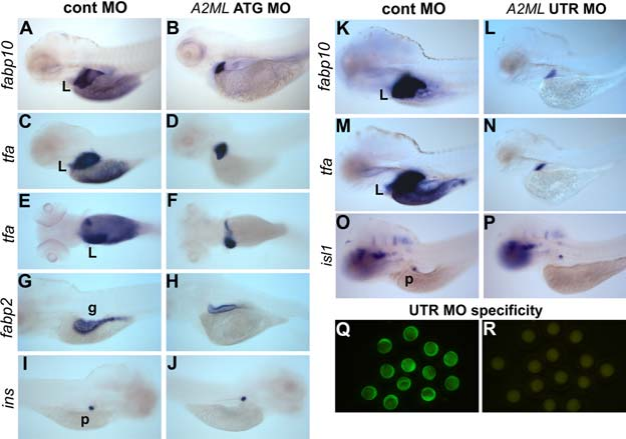
Whole mount in situ hybridization of *A2ML* knock-down embryos. The indicated MOs were injected and embryos were stained at 5dpf A–J or 6dpf K–P. A, B, K, L. Lateral views of *fabp10-*stained embryos. C–F, M–N. Lateral (C, D, M, N) and dorsal views (E–F) of *tfa* expression. G–H. Lateral views of *fabp2* expression. I–J. Lateral view of *insulin* expression, and O–P. *islet1* expression in the pancreas. Q–R. Specificity test using the UTR MO at the 3-somite stage; see Fig. 2K–L.

This effect on liver formation was visualized with the aid of molecular markers at five to six dpf in Fig. 4. The expression domains of liver specific markers such as *intestinal fatty acid binding protein 10 (fabp10)* [23] and *tfa* were greatly reduced after injection of *A2ML* UTR or ATG MO (Fig. 4A–F and 4K–N; see 4Q, R for UTR MO specificity). A similar effect was also seen with the liver marker *cp* (data not shown). The liver and pancreas form as protrusions from the gut primordium during early development [24].

We also monitored possible effects of A2ML MO injection on blood vessel formation in the liver by inspecting fliGFP transgenic animals, and found no substantial defect in blood vessel formation (Fig. S2A–B).

Because of the close relationship in the formation of these organs we also tested whether *A2ML* MOs affect the formation of the gut and pancreas with the aid of specific markers for these tissues. Using the *fabp2* as a marker [18], we find that the gut appears somewhat reduced in size in 5 dpf embryos (Fig. 4H). A reduction in gut size can also be seen by direct inspection of *A2ML* MO-injected embryos (Fig. 3), but the effect of A2ML knock-down on gut formation appears to be less intense than the effect on the liver. The goblet cells in the developing intestine appeared to develop normally in A2ML knockdown embryos (Fig. S2C–D). Expression of *fabp10* and *tfa* in the yolk region was completely lost after *A2ML* MO injection (Fig. 4C–F and 4K–N), and these embryos failed to resorb their yolk and to inflate a swim bladder, defects that were effectively rescued by co-injection of mRNA (Fig. 3).

It is not known whether persistence of the yolk mass impedes gut development in these embryos or reduced gut development is the cause of poor yolk utilization after knock-down of A2ML expression. Proper lipid utilization is required for yolk absorption and normal development in zebrafish [25], and the liver has a major role in lipid metabolism [26]. Thus, failure of proper liver growth in the A2ML-deficient embryos may be an important cause of poor yolk resorption and utilization. In contrast to the effects on liver and gut formation, development of the pancreas appears unaffected by *A2ML* MO injection, as seen with the aid of in situ hybridization with *insulin (ins)* [27] at 5 dpf (Fig. 4I–J), and *islet1 (isl1)* [28] at 6 dpf (Fig. 4O–P). Likewise, exocrine pancreas cells, observed using immunoreactive carboxypeptidase A, develop normally in *A2ML* MO injected embryos (Fig. S2E–F). Thus, A2ML is required for liver growth and development and for gut development to a lesser degree, but does not appear to have a role in pancreas formation.

### A2ML is required for liver cell proliferation and tissue differentiation

**Figure 5:**
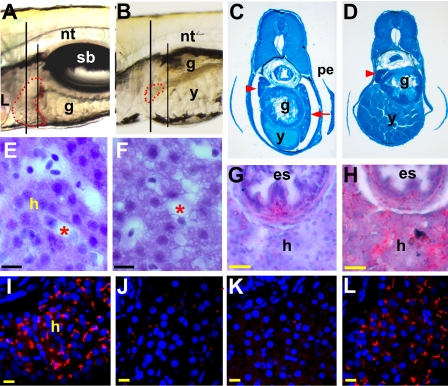
A2ML knock-down phenotypes in the liver. Embryos injected with cont MO (A, C, E, G, I), *A2ML* ATG MO (B, D, F, H, J), *A2ML* UTR MO (K), and embryo rescued by co-injection of ATG MO and mRNA (L) were analyzed at 5.5 dpf. (C–F) plastic sections, (G–L) cryosections. A–B Lateral live images in the area of the liver (indicated by red dotted line); the short vertical line indicates the plane of section for C and D, the long line for E–L. C–D Methylene blue stain. Red arrowhead points to pancreas, red arrow to liver (in C). E–F Hematoxylin and Eosin (H&E) stained liver section. Asterisks are sinusoids. G–H Lipid staining of liver using Oil-Red O for cont MO (G) and *A2ML* ATG MO-injected embryos (H). I–L Confocal images for P-glycoprotein IHC; either MO eliminates P-glycoprotein staining (J and K), while mRNA coinjection rescues staining (L). Scale bars represent 10 µm in E–F and 50 µm in G–L. g, gut; h, hepatocytes; L, liver; n, notochord; pe, pectoral fin; y, yolk.

To probe further into the nature of the inhibition of liver formation after A2ML knock-down we examined embryo sections by histologic techniques. Methylene blue-stained sections of embryos at 5.5 dpf further illustrate the morphological differences between control and A2ML knock-down embryos (Fig. 5C,D). The pancreas was not affected by *A2ML* ATG MO injection, while the yolk mass is prominent in the knock-down embryo compared to the control MO-injected embryo (Fig. 5C,D). While pancreas and gut cross-sections are similar to controls at this stage, the liver is greatly reduced and in fact is not present in the section shown in Fig. 5D. An enlarged section of the liver shows reduced density of nuclei and increased vacuolization in the A2ML-deficient liver (Fig. 5E,F). A deficit in lipid metabolism is also suggested by the greatly increased staining with Oil Red O in the remaining liver tissue of these embryos (Fig. 5G,H). Lack of liver differentiation is apparent in the essentially total loss of P-glycoprotein staining in the livers of embryos injected with the *A2ML* ATG MO or UTR MO (Fig. 5J,K); this effect was rescued by injection of *A2ML* mRNA (Fig. 5L).

**Figure 6:**
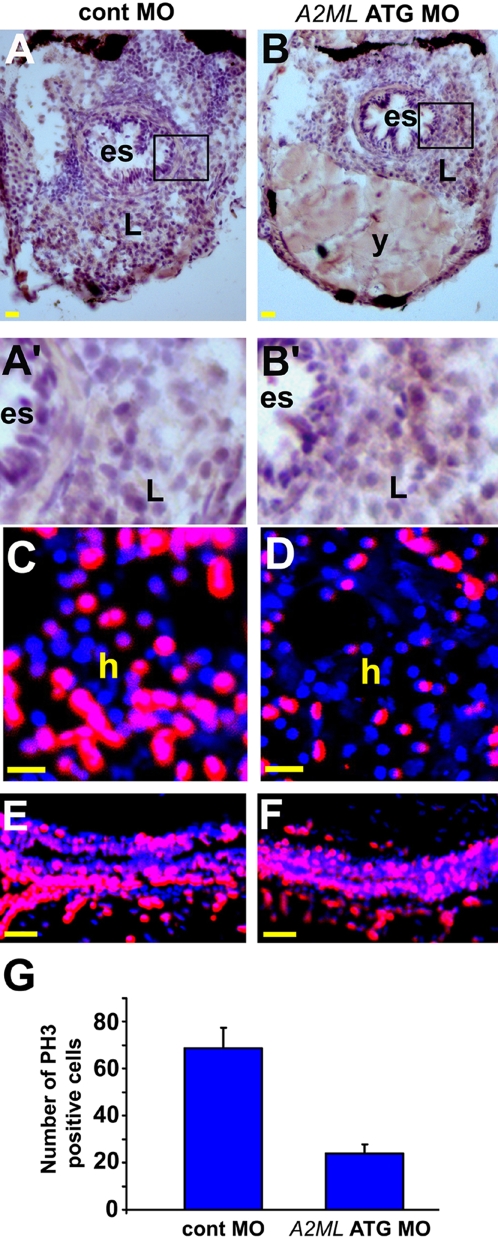
TUNEL and PH3 staining in *A2ML* ATG MO-injected embryos. All embryos were at 5 dpf. A–D are transverse section, and E–F sagital section. A–B′ TUNEL staining of cont MO-injected embryo (A), and knock-down embryo (B). Areas in open square in A and B are magnified in the A′ and B′. C–D Confocal images of sections stained with anti-PH3^ser10^ antibody. E–F PH3 staining in the intestine in cont MO (E) and in *A2ML* ATG MO-injected embryos (F). G Quantification of PH3-positive liver cells. Scale bars in (A and B) 20 µm, and in (C–F) 50 µm. es, esophagus; L, liver; y, yolk.

The great reduction in liver size together with the cellular changes seen in Figures 5E and 5F could be explained by an increase in cell death, a decrease in proliferation, or both. Apoptosis was not increased in *A2ML* MO-injected embryos compared to controls (Fig. 6A–B′). In contrast, proliferation of hepatocytes as tested by anti-phospho histone 3 (PH3) antibody staining showed a clear reduction in A2ML knock-down embryos (Fig. 6C,D and G). In contrast, proliferation of intestinal cells was not obviously compromised in these embryos at the same stage (Fig. 6E–H). It is possible that proliferation in the gut decreases during later development as we observe a reduction in the size of the gut in A2ML knock-down embryos starting at a later stage than for the liver. Thus, inhibition of A2ML expression leads to reduced proliferation in the liver primordium and inhibits differentiation of those cells that remain in the diminished organ.

### Concluding remarks

A2M family proteins are major products of the liver. We have isolated the gene encoding a member of this family, A2ML, from zebrafish and shown that it is expressed in endodermal precursors and subsequently in the liver. Inhibition of A2ML expression with the aid of antisense MOs led to strong reduction in the size of the developing liver without any effect on the formation of the pancreas. This observation is of interest in the sense that a protein normally thought of as a product of the liver proves to be required for liver formation, specifically for cell proliferation in the liver rudiment. These results suggest a relationship between physiology and development in liver organogenesis.

The relationship of zebrafish A2ML to members of the protein family in other vertebrates is difficult to evaluate, mostly because of an incomplete understanding of orthology relationships. Functions and possible disease relationships of the prototypical A2M has been studied extensively in humans (see Introduction), but knockout of the apparent ortholog in the mouse is viable and fertile [10], [11]. The protein we describe in zebrafish appears more closely related to human A2ML (Fig. 1K), a protein discovered more recently and described as a factor with a role in epidermis [29]. Based on existing information, human and zebrafish A2ML have no functional communality, but neither protein has been studied exhaustively to date. The group of A2ML proteins also includes *Xenopus* Panza (Fig. 1K) about whose function little is known, but no similar mouse protein has been reported. A search of sequence data bases has not revealed a zebrafish gene more closely related to the prototypical A2M, but the zebrafish genome is not yet complete and the issue thus remains unresolved. Thus, the evolutionary and functional relationships in the A2M protein family remain to be elucidated by future work.

## Materials and Methods

### Zebrafish Maintenance

The *ef1a* transgenic line, *Tg(XlEef1a1:GFP)s854/+*, was obtained from Zebrafish International Resource Center (ZIRC. http://zebrafish.org/zirc/home/guide.php) and *fli*-gfp transgenic zebrafish were obtained from Dr. Brant Weinstein (NICHD/NIH).

### Isolation of Full-length *A2ML*

The full-length *A2ML* sequence was completed by 5′- and 3′-RACE (BD Biosciences Clontech), using 5′-GCAGCGGTGAAACTCTTGCTCAGCATTC-3′ (5′-RACE) and 5′-CTTCACTGTCAAATACGATG GGCCAGAG-3′ (3′-RACE). Subcloning into pCS2^+^ used primers 5′-GAATTCGTCATCATGGCTCTGAA TGTTAGC and 3′-CTCGAGTTAATT GATGTTCTTCAGTGCTTC, introducing 5′ EcoRI and 3′ XhoI sites. Amino acid comparisons and phylogenic tree analysis were carried out with DNASIS MAX version 2.0 (MiraiBio, Hitachi software).

### In Situ Hybridization

Whole-mount in situ hybridization with alkaline phosphatase-based single or double color staining has been described [30]. Double fluorescent in situ hybridization (FISH) was performed as described [31]. The green color detection used the tyramide signal amplification Kit with horseradish peroxidase, and the red color was Cy3 (PerkinElmer).

### Microinjection of *A2ML* RNA and Morpholinos

*A2ML* ATG MO (5′-ACAACAGCTAACATTCAGAGCCATG-3′) and UTR MO (5′-CAGAGCCATGATGACGAGTGTCCAG -3′) were synthesized by Gene Tools. The injection doses were: cont MO, 10 ng; ATG MO, 7 ng; UTR MO, 2 ng; all were injected into one-cell stage embryos. For rescue, 30 pg of *A2ML-eGFP* RNA was used; the same level was used for specificity tests.

### Histology

Sections were cut after embedding in JB-4 plastic (Polysciences) at 7 µm, or in OCT medium for cryosectioning (Tissue-Tek) at 10 µm for immunohistochemistry (IHC). Embryos were fixed in 4% paraformaldehyde for 2 hours at 4°. For IHC, sections were soaked for 2 min in acetone, followed by 30 min in 10 mM sodium citrate (pH 6.0) in PBS (0.1% Triton X-100). Blocking was done with 2% goat serum in PBS-Tween 20 for 30 min at room temperature. Methylene blue staining has been described [30]. IHC was performed as follows: anti-PH3^ser10^ at 1∶500 (Upstate), anti P-glycoprotein at 1:300 (Abcam), anti-bovine carboxypeptidase A at 1∶500 (Rockland Inc) [32]. Fluorescein conjugated wheat germ agglutinin (Vector lab) [33] was diluted in 1xPBS at 1∶100 and incubated overnight at 4°. Alexa Fluor 488-conjugated anti-mouse IgG and Alexa Fluor 568-conjugated goat anti-rabbit IgG were used as secondary antibodies (Invitrogen). TUNEL reagents were from Invitrogen, and sections were counter stained with Gill's hematoxylin. Lipid staining of cryosections used 0.5% Oil-red O (Sigma) in propylene glycol. Confocal imaging was done with a Zeiss LSM 510 laser-scanning confocal microscope.

## Supporting Information

Figure S1 Amino acid comparison of A2ML family. Amino acid comparison of α2ML family. A. Schematic representation of amino acid sequence comparison between species. The percentage identity to zebrafish α2 macroglobulin-like is given for N-terminal (N), middle (M), and C-terminal (C) regions, and for the entire proteins to the right. B. The bait regions (bold underline) are compared. C. The highly conserved thio ester region. A glycine and glutamine (asterisks) represent essential amino acids that form thio ester bonds. D. C-terminal highly conserved target binding domain (CHB) (bold underline). The accession number for A2M family are carp A2M-1 (AB026128); Xenopus laevis Panza (DQ080115); Xenopus laevis EDD (L63543); Human A2M (BC040071); Human A2ML1 (NM144670); Mouse A2M (BC072642).
(5.97 MB TIF)

Figure S2 Development of blood vessels, goblet cells, and exocrine pancreas. Embryo stages are 5 dpf for A–D, and 4dpf for E–F. A–B. Blood vessel formation of control MO (A) and A2ML MO (B) injected embryos as visualized in fli1-gfp Tg embryos. C–D. Confocal images of goblet cells in intestine were obtained using fluorescein-conjugated wheat germ agglutinin in control MO (C) and A2ML MO (D) injected embryos. E–F. Confocal images of immunoreactive carboxypeptidase A showing exocrine cells in the pancreas in cont MO (E) and A2ML MO (F) injected embryos. L, liver.
(8.65 MB TIF)
