# Supplementary figures and images for: Alpha2 Macroglobulin-Like Is Essential for Liver Development in Zebrafish
Source: PLoS One. 2008 Nov 17;3(11):e3736. doi: 10.1371/journal.pone.0003736 (PMC2581608; doi:10.1371/journal.pone.0003736)

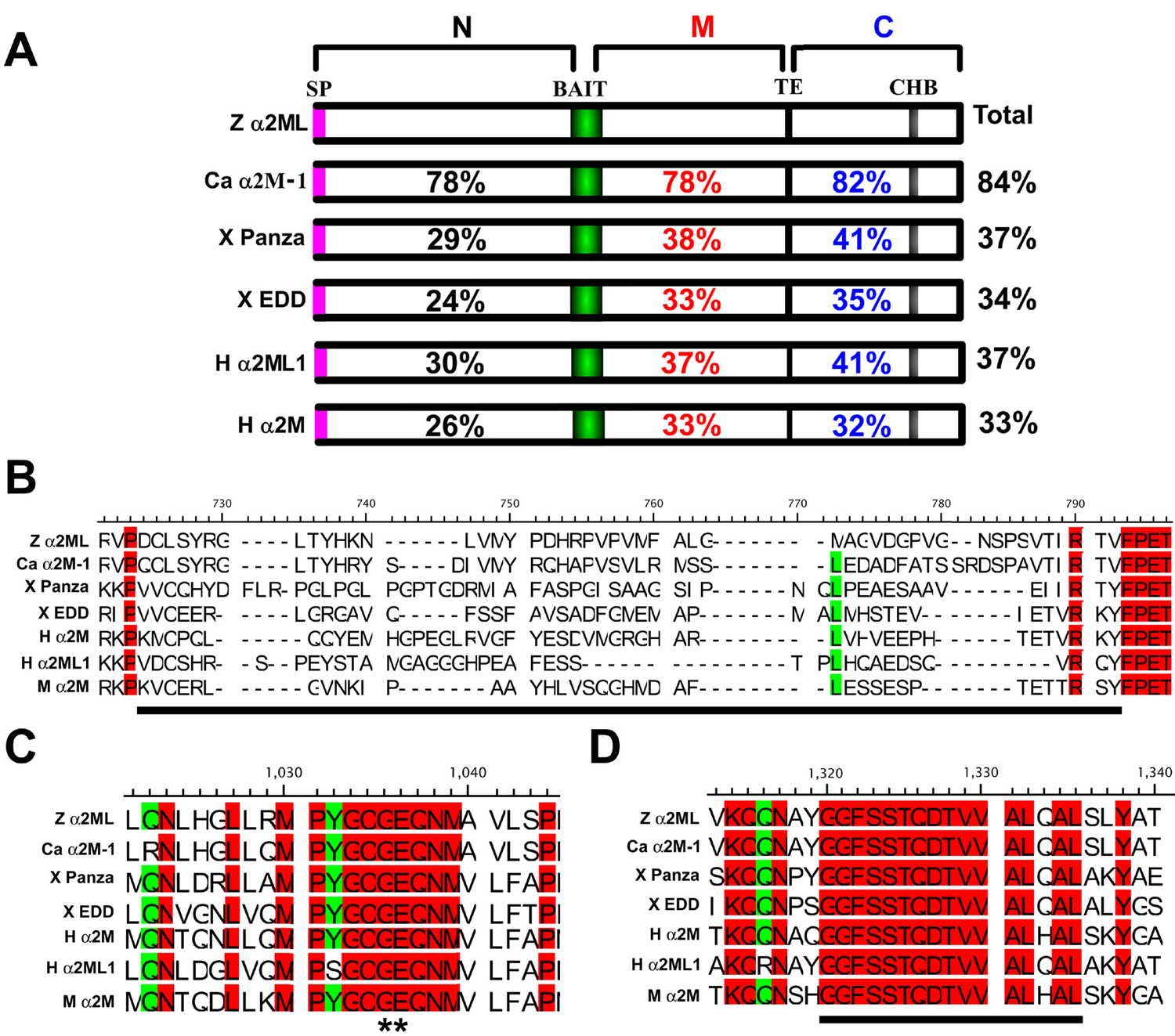

Supplement: Figure S1 — Amino acid comparison of A2ML family. Amino acid comparison of α2ML family. A. Schematic representation of amino acid sequence comparison between species. The percentage identity to zebrafish α2 macroglobulin-like is given for N-terminal (N), middle (M), and C-terminal (C) regions, and for the entire proteins to the right. B. The bait regions (bold underline) are compared. C. The highly conserved thio ester region. A glycine and glutamine (asterisks) represent essential amino acids that form thio ester bonds. D. C-terminal highly conserved target binding domain (CHB) (bold underline). The accession number for A2M family are carp A2M-1 (AB026128); Xenopus laevis Panza (DQ080115); Xenopus laevis EDD (L63543); Human A2M (BC040071); Human A2ML1 (NM144670); Mouse A2M (BC072642). (5.97 MB TIF) [file pone.0003736.s001.tif]

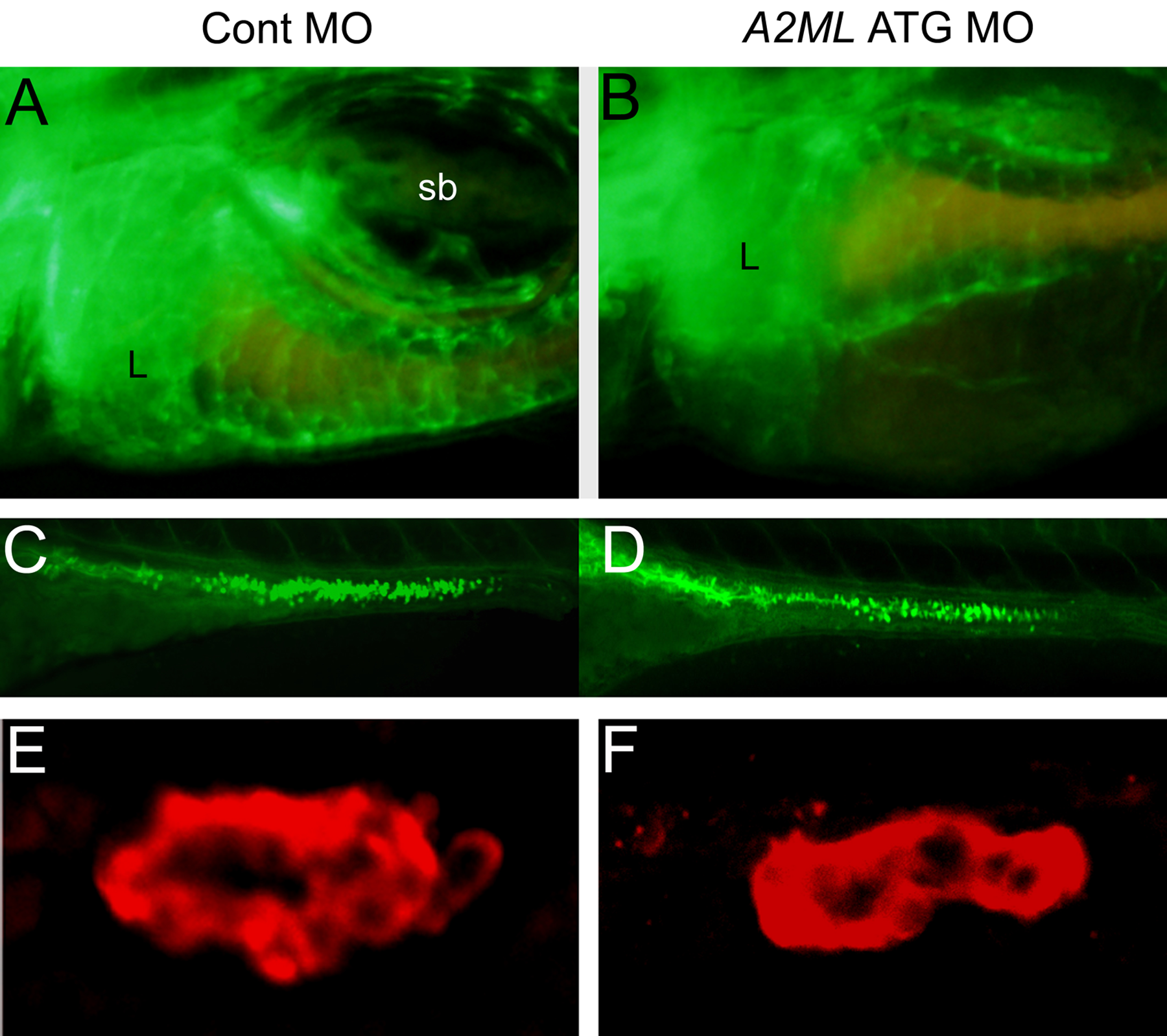

Supplement: Figure S2 — Development of blood vessels, goblet cells, and exocrine pancreas. Embryo stages are 5 dpf for A–D, and 4dpf for E–F. A–B. Blood vessel formation of control MO (A) and A2ML MO (B) injected embryos as visualized in fli1-gfp Tg embryos. C–D. Confocal images of goblet cells in intestine were obtained using fluorescein-conjugated wheat germ agglutinin in control MO (C) and A2ML MO (D) injected embryos. E–F. Confocal images of immunoreactive carboxypeptidase A showing exocrine cells in the pancreas in cont MO (E) and A2ML MO (F) injected embryos. L, liver. (8.65 MB TIF) [file pone.0003736.s002.tif]
